# A low cost and open access system for rapid synthesis of large volumes of gold and silver nanoparticles

**DOI:** 10.1038/s41598-021-84896-1

**Published:** 2021-03-08

**Authors:** Alex Ross, Marcelo Muñoz, Benjamin H. Rotstein, Erik J. Suuronen, Emilio I. Alarcon

**Affiliations:** 1 Division of Cardiac Surgery, University of Ottawa Heart Institute, 40 Ruskin street, Ottawa, ON K1Y4W7 Canada; 2 Biochemistry, Microbiology and Immunology, University of Ottawa, 451 Smyth Road, Ottawa, ON K1H8M5 Canada; 3 Molecular Imaging Probes and Radiochemistry Laboratory, University of Ottawa Heart Institute, 40 Ruskin street, Ottawa, ON K1Y4W7 Canada

**Keywords:** Materials science, Nanoscale materials, Nanoparticles

## Abstract

Rapid synthesis of nanomaterials in scalable quantities is critical for accelerating the discovery and commercial translation of nanoscale-based technologies. The synthesis of metal nanogold and silver in volumes larger than 100 mL is not automatized and might require of the use of harsh conditions that in most cases is detrimental for the production of nanoparticles with reproducible size distributions. In this work, we present the development and optimization of an open-access low-cost NanoParticle Flow Synthesis System (NPFloSS) that allows for the rapid preparation of volumes of up to 1 L of gold and silver nanoparticle aqueous solutions.

## Introduction

The application of nanoparticles has rapidly emerged in a variety of fields spanning from heavy metal ion detection^1–3^, drug delivery^4^, nuclear targeting^5^, biosensing^6,7^, and microscopy contrast enhancement^8^ to transfecting agents^9^. The process of nanoparticle functionalization*,* or capping, with inorganic^10^ or biological^11^ capping agents is key in facilitating these applications as it allows for control over properties such as nanoparticle size, biomolecular recognition, and can even impart advanced characteristics such as pH-dependent size control^12^. Furthermore, the inclusion of nanoparticles in biomimetic matrices can provide a variety of properties such as antimicrobial ability^13,14^ and electrical conductivity^15^. However, nanoparticle synthesis has intrinsic batch-to-batch variability in particle diameter polydispersity and surface composition that contributes to discrepancies in nanoparticle performance and activity^16^. Metal nanoparticle synthesis is normally conducted using chemical reduction in batch reactor setups^17^. The photochemical reduction of metal ions to produce nanoparticles is an interesting alternative route for producing nanostructures from metals due to its simplicity and compatibility with a wide variety of capping agents. Both gold and silver nanoparticles can be synthesized from the photoinitiated reduction of HAuCl_4_ via the Norrish Type I cleavage reaction of Irgacure-2959^18,19^ in an aqueous solution^20^. However, scaling up photochemical preparation of nanoparticles remains a challenge, particularly when considering limited UVA light penetration in large containers.

Flow-based chemistry is becoming increasingly popular due to its advantages over batch chemistry such as reduced batch-to-batch variability^21^. Previous work on flow irradiation suggests that particle size and polydispersity can be adjusted simply by altering the flow rate or irradiance intensity^22^. However, reactor fouling remains a serious problem with flow-based nanoparticle synthesis. A recent approach to preventing fouling can be seen in the work of *Monbaliu et* al^23^, who used a specific, tightly pH controlled gold citrate formulation to allow large scale synthesis in commercial photoreactors. However, this approach is limited by the significant cost of a commercial photoreactor and the use of one specific capping agent along with sodium ions which can contaminate metal nanoparticles.

Herein, we report a low-cost (~ 200 CAD), open access system called the Nanoparticle Flow Synthesis System (NPFloSS) that utilizes LED-UVA light sources to generate a continuous flow of nanoparticles. NPFloSS produces batches of both gold and silver nanoparticles within minutes, which is much faster than the normal batch photoreactor. Furthermore, the use of individual LEDs considerably lowers the cost, increases the system lifetime, and reduces physical space requirements when compared to commercial photoreactors. A general formulation is used that can incorporate any desired capping agents without contaminating metal ions. Furthermore, Tween-20 surfactant is presented as a novel method for anti-fouling in the context of flow nanoparticle synthesis.

## Results

### NPFloSS development

**Figure 1:**
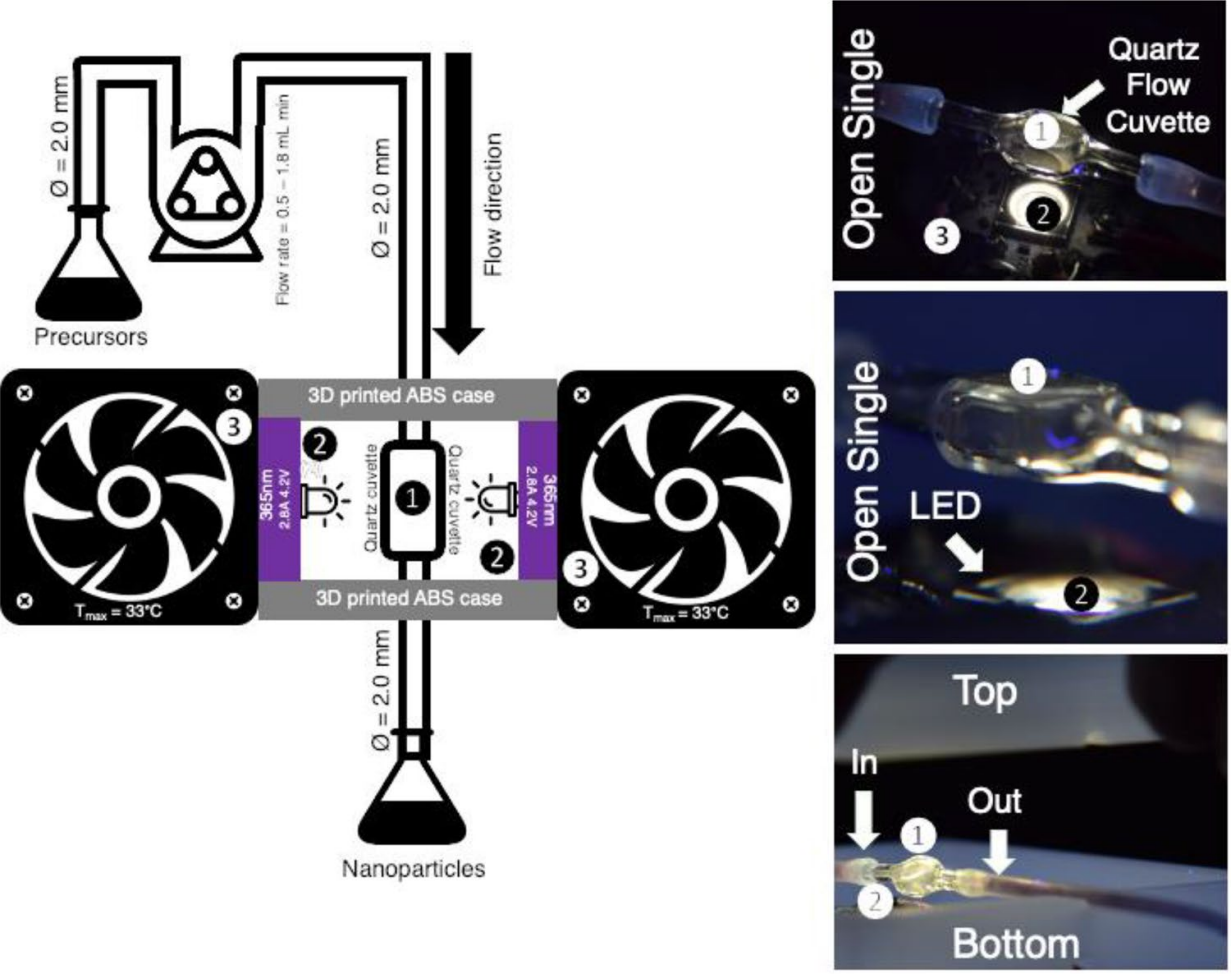
Design of nanoparticle flow synthesis system. Left: Schematic representation for the Nanoparticle Flow Synthesis System (NPFloSS). Right: Actual pictures of the NPFloSS highlighting some key components of the design. The NPFloSS is composed of three simple main components: (1) a UV-transparent quartz cell with connected by tubing, (2) two 365 nm UVA LEDs as a photon source, and (3) two heat sinks for heat management. The part list information and assembly instructions for this system are available at no cost in the ESI of the article.

The NPFloSS consists of three main simple components: (1) a flow line driven by a peristaltic pump containing a UV transparent quartz cell (recycled from a decommissioned HPLC), (2) two 365 nm UVA LEDs, and (3) two heat sinks (Fig. 1). Assembly instructions and additional details are available in the tutorial Video S1 and in the ESI. In the NPFloSS, the input tubing leads to a peristaltic pump that flows the reactants into a quartz flow cell, which is housed within a 3D printed compact UVA irradiation system containing two LEDs to provide high intensity irradiation (up to 14 mW/cm^2^). The LEDs’ temperature is maintained using Alpine GT CPU fans. After UVA exposure, the products are generated and flow into the output collector. The 3D printed compact UVA irradiation case allows for benchtop setup and reproducibility while removing the need for additional components or holders. The system can be continuously run to generate + 100 mL of product nanoparticle solution an hour or 1.0 L of product if left overnight.

### NPFloSS optimization and nanoparticle synthesis

Initial attempts at synthesis showed that in order to produce quality nanoparticles at an acceptable rate, high light intensity was critical. As such, two powerful LEDs were installed, and the effect of different light intensities evaluated (see Figure S1). The highest intensity (≈ 14 mW/cm^2^) resulted in the best outcome for producing nanoparticles with the most intense plasmonic absorption at their respective maxima absorption (see Figure S1A). As higher intensity seemed beneficial, the LEDs were run at their maximum recommended operating current at 365 nm to produce a light dosage of 37 mJ/cm^2^ for gold and 128 mJ/cm^2^ for silver.

At the optimal light dosage, the LED circuit board was found to stabilize at an operating temperature of 33 °C while the irradiated solution inside the quartz cell reached a temperature of 31 °C after 120 s (Figure S2). Note that the time the solution is exposed to 31ºC is less than 3s for gold and 10s for silver, which is enough time for the metal reductions to take place. Furthermore, maintaining the LED at this temperature allows for extending the LED lifespan.

**Scheme 1:**
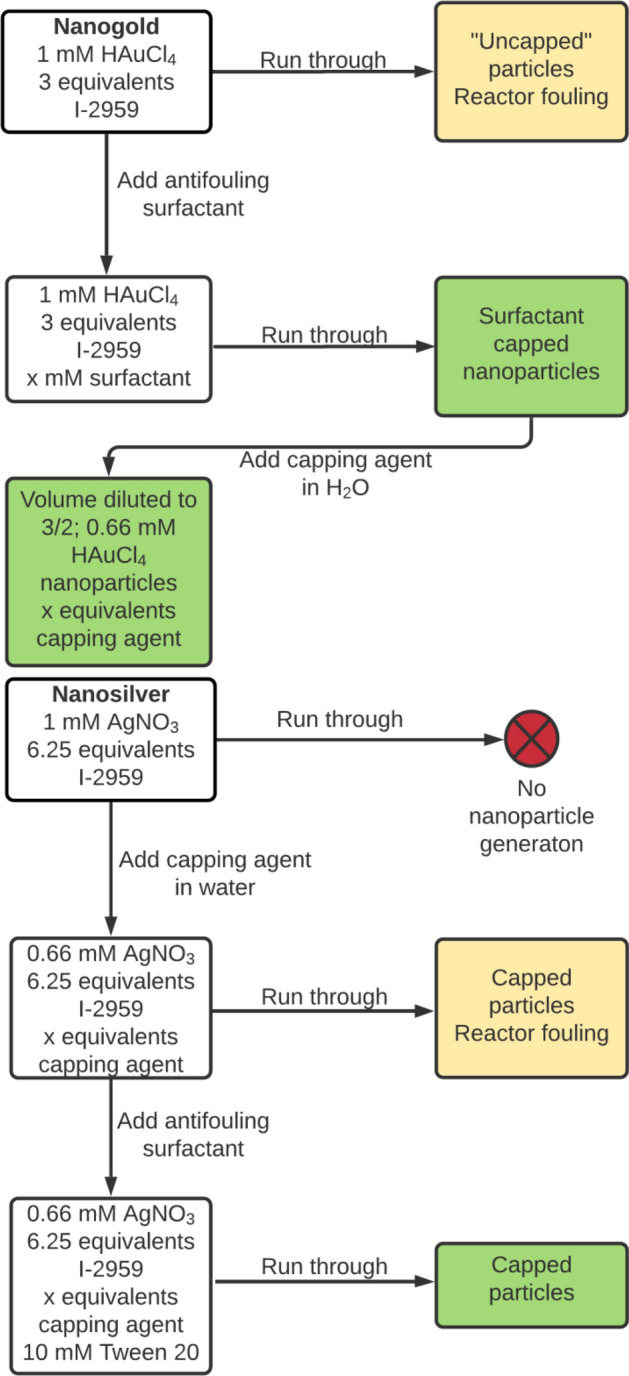
Optimized schematic representation for operation of the Nanoparticle Flow Synthesis System (NPFloSS). For nanogold (top) or nanosilver (bottom).

At this stage of the NPFloSS development, nanoparticles could be made but reactor fouling was a major issue, which led to increasing the risk for line clogging and system failure (see Figure S3). Attempts to reduce fouling were made by modifying several characteristics such as flow rate, pH, temperature, and capping agent. Ultimately, the use of 10 mM Tween-20 surfactant added to the flow mixture was found to be effective to prevent fouling. Tween-20 is a mild surfactant capping agent^24^ that can be displaced by other capping agents and shows good compatibility with biomolecules^25^. Tween-20′s anti-fouling action comes from its dual behaviour as both a surfactant and a capping agent that causes forming nanoparticles to better interact with the aqueous environment and remain in solution instead of building-up on the quartz. The protocol used for preventing fouling is shown in Scheme 1 (for further details see ESI).

**Figure 2:**
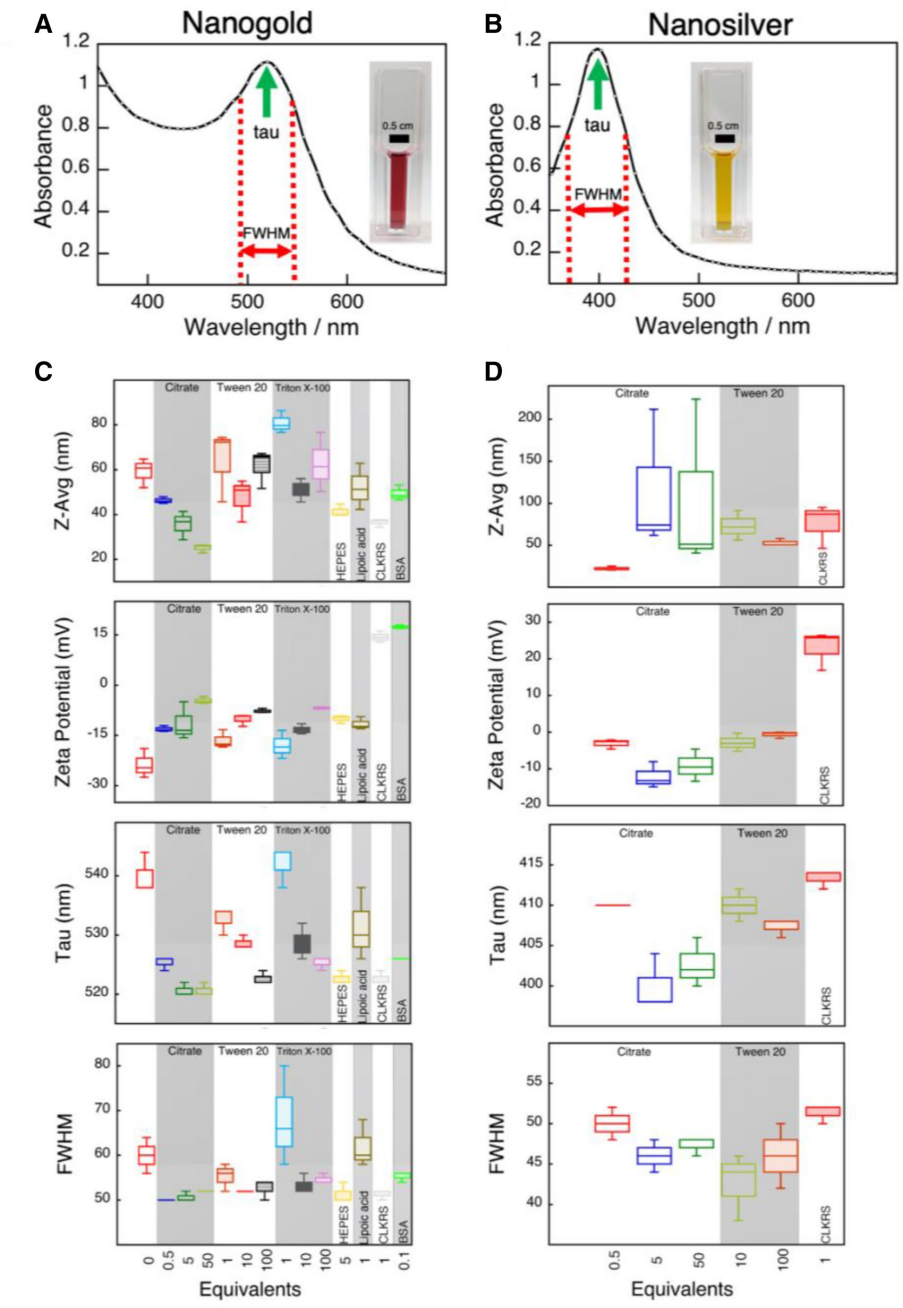
Colloidal properties for nanogold and nanosilver solutions prepared using NPFloSS. NPFloSS allows for rapid nanogold and nanosilver synthesis. Absorption spectra for nanogold (**A**) and nanosilver (**B**) aqueous colloidal solutions prepared using NPFloSS; gold nanoparticles capped with bovine serum albumin and silver nanoparticles capped with CLKRS peptide. The spectra illustrate representative examples for the determination of tau, the wavelength of maximal plasmon band absorbance, and full width at half maximum (FWHM) of the plasmon band. (**C,D**) from top to bottom: Hydrodynamic size, zeta potential, Tau, and FWHM values for gold (**C**) and silver (**D**) nanoparticles prepared in the presence of different protecting agents (see Scheme 1 for synthesis protocol). For each different equivalency of protecting agent, three batches were produced and measured in triplicate. Values in plots (**C,D**) are represented as box plots where the box encloses 50% of the data, upper and lower quartile, with the median value of the variable displayed as a line inside the box. The bars extending from the top and bottom of each box mark the minimum and maximum values within the data set that fall within an acceptable range. Sample size is n = 3 in all cases. See Table S3 for statistical analysis.

With the operating conditions optimized (see Scheme 1), the stability of both “uncapped” and citrate capped gold nanoparticles was tested. Citrate capped nanoparticles showed better stability over a month than uncapped gold nanoparticles (Figure S4). In general, all nanoparticles grew for ~ 24 h after synthesis and those with less stable capping agents continued to slowly grow thereafter. This confirms previous reports of nanoparticle growth after synthesis^26,27^. Representative absorption spectra for nanogold and nanosilver colloidal solutions are showed in Fig. 2A,B, respectively. Representative absorption spectra for the different nanogold and nanosilver particles are included in Figures S5 and S6. The effect of citrate as a capping agent in the case of gold showed a net decrease in the hydrodynamic size from ≈ 60 nm for uncapped particles to ≈ 25 nm for the highest concentration (50 eq) of citrate (Fig. 2C, *p* < 0.05 t-test, see Table S3 for stats summary). Zeta potential values remained mostly unchanged, while the maximum absorption wavelength in the visible (tau) blue shifted by ≈ 30 nm to shorter wavelengths at the highest citrate concentration used. This was also accompanied by a reduction on the spectra broadness or full width at half max (Fig. 2C). TEM analysis revealed that nanoparticle sizes ranged from ≈ 60 to ≈ 9 with estimated polydispersity (PDI) mostly to be in the monodisperse range < 0.1 as showed in Table S4.

**Figure 3:**
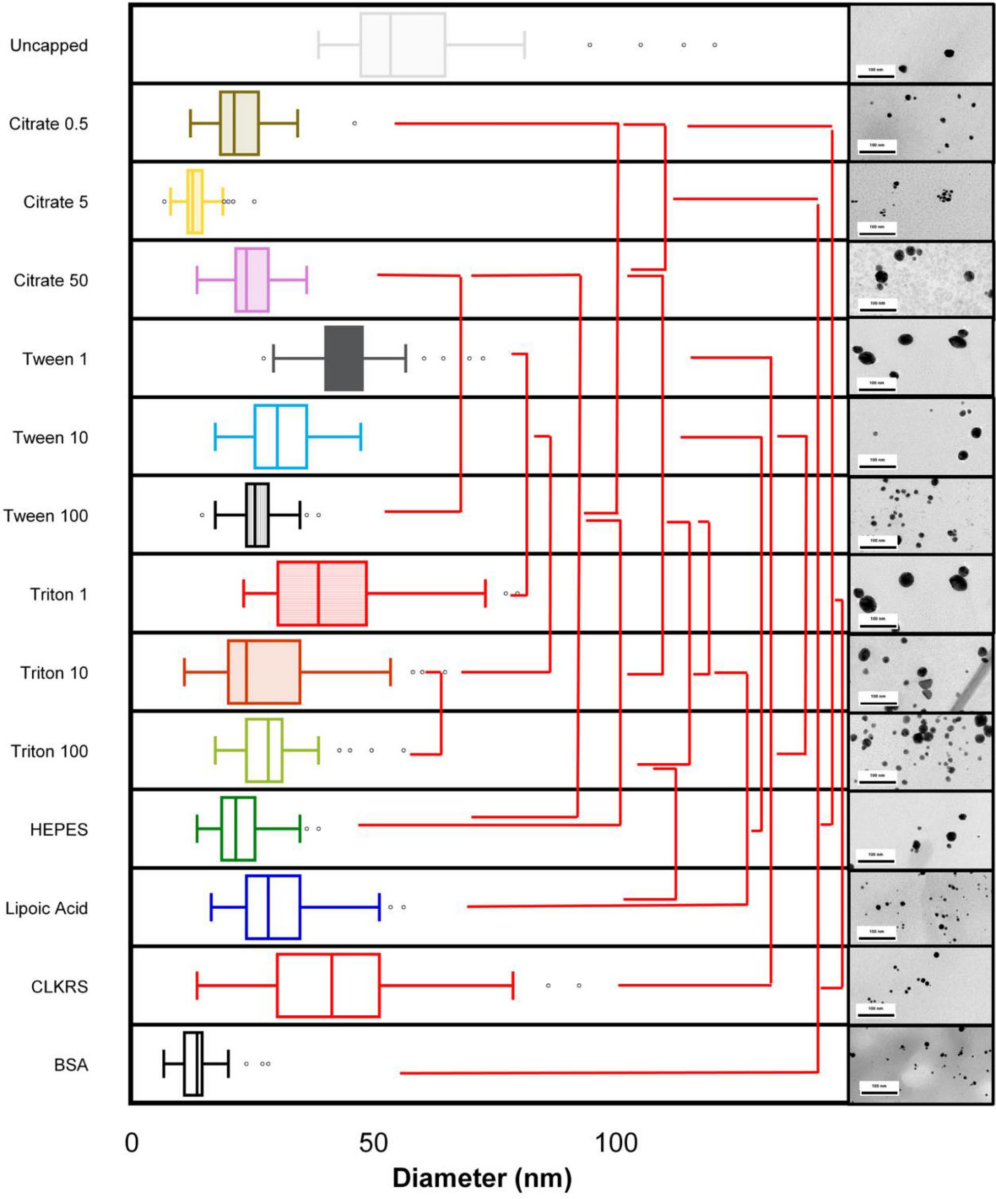
Transmission electron microscopy images for nanogold and nanosilver prepared using NPFloSS. Nanogold TEM measurements corroborate DLS measurements with regards to particle size for nanoparticle samples made by NPFloSS. The type of nanoparticle, capping agent used, and number of equivalents for the capping agent are presented to the left side of each histogram. Each histogram represents 30–100 individually measured nanoparticles. Representative TEM images of the nanoparticles are shown to the right of each histogram along with a 100 nm scale bar. Values in the figure are represented as box plots where the box encloses 50% of the data, upper and lower quartile, with the median value of the variable displayed as a line inside the box. The bars extending from the top and bottom of each box mark the minimum and maximum values within the data set that fall within an acceptable range. *p* values are calculated by one-way ANOVA using Holm's multiple comparison analysis. Bars in red indicate no statistically significant differences (see Table S5).

Nanoparticle sizes measured by TEM showed a statistically significant reduction of the nanoparticle sizes from 62 to 25 nm (*p* < 0.05, values were determined by one-way ANOVA using Holm's multiple comparison, see Table S4 for TEM stats summary), at 0 and 50 citrate equivalents (Fig. 3). Figures S7 and S8 contain representative TEM images for the prepared nanogold and nanosilver particles. Using nanogold particles prepared with five equivalents of citrate as a capping agent, we evaluated the batch-to-batch reproducibility for our system, see ESI for details. TEM and DLS measurements of the nanoparticles indicate that NPFloSS produces reproducible batches, see Tables S6 and S7.

For the case of nanosilver, uncapped synthesis was not possible. Fine tuning of the flow rate for synthesizing nanosilver was required (see ESI and Materials and Methods for details). Nanosilver was stable with negative zeta potentials when using citrate as a capping agent (Fig. 2B,D), with increasing particle hydrodynamic sizes that became considerably polydisperse at 5 and 50 equivalents of citrate (Fig. 2D). TEM experiments showed that nanosilver was statistically larger (> 5 nm, determined by one-way ANOVA using Holm's multiple comparison) for the 5 and 50 equivalents of citrate compared to the 0.5 equivalents group (Figure S9, see Table S8 for statistical analysis). Nanosilver at 0.5 equivalents of citrate displayed a wide plasmon band that was red shifted as equivalents increased from 5 to 50 as would be expected from the increasing particle size.

For Tween-20 capped nanogold there were no significant changes in the hydrodynamic sizes. However, there is a light increase in the zeta potential values of about ≈ 5 mV at 1 and 10 equivalents of the surfactant. Particles with neutral (− 30 to + 30 mV) zeta potentials are commonly used in biomedical applications^28–30^. Surfactants such as Tween-20, although neutral, provide stability to nanoparticles^25^.

In contrast, there is a marked blue shift of ≈ 10 nm in tau at larger Tween-20 equivalent numbers (Fig. 2C). Full width at half max (FWHM) remained mostly unchanged. TEM measurements of individual nanoparticles prepared with 10 or 100 equivalents of Tween-20 showed significantly different values for the mean size (*p* < 0.05, determined by one-way ANOVA using Holm's multiple comparison, see Table S2. As for nanosilver, Tween-20 did not change the nanoparticle hydrodynamic sizes. Similar data was observed for zeta potential, maximum absorption and FWHM (Fig. 2D). TEM images of the 10 and 100 equivalent Tween-20 capped silver nanoparticles also indicated that size was similar between groups (Figure S9 and Table S6).

When Triton was used as a capping agent, at 10 equivalents there was a decrease in the hydrodynamic sizes to values closer to those observed for the 1 equivalent of Triton (Fig. 2C). For tau, the most pronounced change was found for Triton, which blue shifted by ≈ 15 nm when increasing the concentration of the surfactant. FWHM values narrowed at 10 and 100 equivalents of the surfactant. The changes in the nanogold hydrodynamic sizes align well with the TEM findings that showed a size decrease from ≈ 42 to 28 nm at 1 and 10 Triton equivalents (see Fig. 3, *p* < 0.05 Table S4 determined by one-way ANOVA using Holm's multiple comparison). Further increasing Triton concentration beyond 10 equivalents did not have a significant effect on the nanoparticle size.

Using smaller molecules such as HEPES, Lipoic acid, and the CLKRS peptide led to the production of stable nanoparticles with hydrodynamic and TEM sizes in the ≈ 10–60 nm range (Figs. 2C, 3). Lipoic acid coated nanoparticles showed a broader absorption spectrum, FWHM, with zeta potential values in the order of − 12 mV, which is considerably lower than the other thiol containing capping agent (CLKRS) that displayed + 15 mV. This aligns well with the reported findings of this short peptide as a superior capping agent for metal nanostructures^31^. Similar trends were observed for nanosilver and CLKRS (Fig. 2D). When using bovine serum albumin (BSA) as a capping agent for nanogold, nanoparticles with sizes in the 50 nm range were produced with strong positively charged surfaces (Fig. 2C), which resembles findings by our group and others in the production of protein capped metal nanostructures^13,14,32,33^. TEM measurements for the BSA coated nanogold indicated considerably smaller nanoparticle diameters of ≈ 30 nm than those measured by dynamic light scattering. These differences might be related to the formation of a protein corona around the nanoparticle when in solution, a phenomenon that does not exist under the experimental conditions of TEM^34^. Furthermore, DLS measurements tend to be larger^35^ due to the dynamic nature of nanoparticles in solution and the measurement of hydration sphere diameter^36^ which includes surface capping molecules as opposed to the dense metallic core measured by TEM.

## Discussion

Flow systems have recently received a lot of attention and as a result systems ranging from simplified to highly automated have been produced to facilitate reagent mixing along with thermal, photochemical, and electrochemical stimulus^37^. The advent of flow photochemical systems has helped solve some of the problems leading to the under-utilization of photosynthetic routes such as specific glassware requirements, scalability, safety, and technical knowledge^38^. Thus, to encourage use of promising photosynthetic techniques, their application should be made as simple and cost-accessible as possible. Due to their wide variety of uses, there is an increasing demand for producing cost-effective methodologies for large scale synthesis of metal nanoparticles. In designing such systems, considerations for having reliable methodologies that encompass rapid capping agent replacement and batch-to-batch reproducibility are key. Flow reactors, such as NPFloSS, provide a cost-effective solution for use in academic or industrial settings.

Reactor fouling is the most common and serious issue when designing flow systems, as the metals tend to deposit on surfaces. In NPFloSS, we used low concentrations of Tween-20 that decreases the contact angle between quartz and water and thus increases the wettability of quartz while reducing interfacial tension^39^. Furthermore, Tween-20 acts as a stabilizing nanoparticle capping agent^40^ while interacting with water through its hydrophilic domains, thus increasing nanoparticle-solution interaction. Through these dual functions, Tween-20 prevented reactor fouling and allowed the system to operate continuously without line blockage. As a biocompatible additive used in food and cosmetics that increases nanoparticle stability in biological media^25^, Tween-20 is a safe addition to biomedical nanoparticle formulations.

NPFloSS’ photochemical reduction methodology may be extended to allow for flow synthesis of other nanoparticle types. Further improvements of NPFloSS could include operation inside a glovebox for synthesis of oxygen sensitive nanoparticles like copper^41^ or its use as an in line system for dual photochemical synthesis of nanoparticles and photocatalysis. Also, the use of a bidirectional inlet system for in situ mixing in the “reaction cell” could be used for the preparation of core–shell nanoparticles.

## Conclusions

We developed, optimized, and validated a low-cost and open access flow reactor for rapid synthesis of gold and silver nanoparticles: the NPFloSS. The practicality of this device allows for rapid synthesis of stable nanoparticles whose surfaces can be readily protected with capping agent molecules such as citrate, Triton X-100, Tween, HEPES, lipoic acid, peptides such as CLKRS, and bovine serum albumin. The open access and low-cost features of NPFloSS make this instrument attractive for use by anyone to expand its nanoparticle synthesis potential to other metals and capping agents.

## Methods

### CAD modeling and 3D printing

All 3D printed components were designed using CAD software (Fusion 360, Autodesk Inc) and printed using an Ultimaker S5 with ABS filament. The instructions and tutorial video for assembling the system are made available in the ESI of this article.

### Other components

2.0 mm Teflon tubing was used to connect all components. A Manostat cassette pump was used to generate flow at a rate of 0.5–1.8 mL/min. Arctic Alpine 64 GT CPU fans were used as a heat sink for two 365 nm LEDs (LZ4-V4UV0R, Mouser) running at 2.8A 4.0 V powered by two adjustable power sources (Yihua PSN-305D). A quartz cell was used as the irradiation chamber.

### Light irradiance calculations

Radiance was directly measured at the flow cell’s position using a Luzchem L-0487 power meter. The obtained reading in lux was converted to W/m^2^ power for 365 nm light using the conversion factor 0.0027. The total light dosage was obtained by multiplying the power by residence time.

### Temperature measurement

A thermal probe was placed in contact with the LED starboard and the temperature was continuously measured until thermal equilibria had been reached and for 10 min thereafter. Solution temperature was measured by flowing water into the quartz cell, turning on the LEDs, and then quickly extruding the water onto a thermal probe after a given time.

### Chemicals

Milli-Q water was freshly prepared using a Barnstead NANOpure II water filtration system. for z. All nanoparticle reactions were performed in an aqueous environment. Irgacure-2959 ([2-Hydroxy-4′-(2-hydroxyethoxy)-2-methylpropiophenone], 98%, Sigma-Aldrich) stock was prepared at a concentration of 10 mM and used as a photoinitiated reducing electron source. HAuCl_4 **⋅**_ xH_2_O (chloroauric acid, 50% basis, Sigma-Aldrich) was used as an aqueous gold source and AgNO_3_ (silver nitrate, 99%, Sigma-Aldrich) as a silver source.

Tween-20 (polyethylene glycol sorbitan monolaurate, ~ 20n, Sigma-Aldrich), sodium citrate dihydrate (99.9%, Fisher), Triton-X 100 (~ 10n, VWR)), HEPES (N-[2-Hydroxyethyl] piperazine-N’-[2-ethanesulfonic acid], 99.5%, Sigma), lipoic acid (98%, Sigma), CLKRS peptide (95%), and bovine serum albumin (98%, Sigma) were used as stabilizing nanoparticle capping agents. Gold nanoparticles were synthesized at a concentration 1 mM and then diluted with solution containing capping agent to 0.66 mM while silver nanoparticles were synthesized at 0.66 mM. After mixing the base reagents (water, I-2959, Tween-20, HAuCl_4_) pH was ~ 5 and after the reaction pH decreased to ~ 2 as measured by pH test strips (Sigma-Aldrich).

### Peptide synthesis

CLKRS peptides were synthesized using the Liberty Blue (CEM) automated microwave peptide synthesizer using N,N-dimethylformamide (DMF, VWR, 99.9%) . Fluorenylmethoxycarbonyl **(**fmoc) protected L-amino acids (99%) were purchased from CEM. To serine-preloaded Wang resin (CEM, 100–200 mesh, 0.3 mmol/g loading), Fmoc deprotection was carried out with 20% piperidine (Sigma, 99%) at 90 °C for 60 s while standard coupling cycles using *N,N′*-diisopropylcarbodiimide **(**DIC, Sigma-Aldrich, 99%) activator and Oxyma Pure (CEM, 99.5%) activator base were run at 90 °C for 120 s. Peptides were removed from the resin and deprotected with 92.5/2.5/2.5/2.5% v/v trifluoroacetic acid (TFA, Caledon, reagent grade)/triisopropylsilane (TIS, Sigma-Aldrich, 98%)/2,2′-(ethylenedioxy) diethanethiol (EDT, Sigma-Aldrich, 95%)/H_2_O at 37 °C for 40 min and then precipitated in -20 °C diethyl ether (Sigma-Aldrich, 98%). Peptides were then dried under gentle N_2_ and purified through reversed phased high-performance liquid chromatography (RP-HLPC). Peptide purity and identity was confirmed via UV/MS. A purity of 95% was determined through UV peak analysis. Mass spectrometry results gave the most abundant peaks of 202.9 [M + 3H +] and 303.8 [M + 2H +] for an experimental mass of 605.6 Da compared with a calculated mass of 605.8 Da (see Figure S10).

### Absorption spectra

To characterize the nanoparticles, a UV absorbance spectrum from 350 to 750 nm was taken using a SpectraMax M2e with 2 nm steps. Three independent samples were prepared for each type of nanoparticle/capping agent. The plotted data in this article corresponds to the mean of those measurements. All samples were measured at a concentration of 0.66 mM.

### Dynamic light scattering and zeta potential measurements

Hydrodynamic sizes and zeta potential measurements were carried out in a Malvern Zetasizer Nano ZS at 20 °C in 1.0 cm pathlength disposable plastic cuvettes. Reported values correspond to the average of three independent batches, each measured in triplicate. All samples had a concentration of 0.66 mM HAuCl_4_ or AgNO_3_ and equivalents of capping to agent to that concentration as reported (*i.e.* 10 equivalents give a concentration of 6.6 mM capping agent).

Replicability DLS experiments were carried out in a Wyatt Technologies DynaPro PlateReader-II at 25 oC in 96 well plates. 10 acquisitions were made for each of 3 batches both 1 day and 7 days after synthesis and are reported as mean ± standard deviation (see S9). All samples had a concentration of 0.66 mM HAuCl_4_ and 5 equivalents (3.3 mM) of citrate capping agent.

### Nanoparticle stability assays

Stability tests for nanogold particles was carried out by measuring the absorbance spectra from 350 to 750 nm in a SpectraMax M2e with 2 nm steps immediately after synthesis (day 0), a day after synthesis (day 1), 7 days after synthesis (day 7), and 28 days after synthesis (day 28).

### Transmission electron microscopy

Samples for electron microscopy were prepared by delivering ~ 10 μL of solution to carbon-coated copper grids (400 mesh) and dried in a vacuum system for three days. Electron microscopy images were taken in a FEI Tecnai F20 G2 FE-TEM, operating in the transmission mode (TEM) at 200 kV. Nanoparticle mean size was calculated from TEM imaging by using ImageJ software^42^ to manually measure 100 individual particles from different areas in the grid. Nanoparticle polydispersity were estimated using PDI = (SD/mean)^2^.

### Statistical analysis

Unless otherwise indicated, *p* values were calculated using t-test or one-way ANOVA in KaleidaGraph 4.5 software. For t-test a *p* < 0.05 was considered as statistically significant. For the one-way ANOVA, a Holm's multiple comparison analysis was performed.

## Supplementary Information

Supplementary Information

## Data Availability

All data generated or analyzed in this study are included in the manuscript and the Supplementary materials. The experimental data that support the findings of this study can be found at no cost 10.6084/m9.figshare.13822775.v1 additional information is available from the authors.
