# Supplementary material for: A low cost and open access system for rapid synthesis of large volumes of gold and silver nanoparticles
Source: Sci Rep. 2021 Mar 8;11:5420. doi: 10.1038/s41598-021-84896-1 (PMC7940392; doi:10.1038/s41598-021-84896-1)
Supplement: Supplementary file 1 — Supplementary Information [file 41598_2021_84896_MOESM1_ESM.pdf]

Supplementary Information (SI) for:

A low cost and open access system for rapid synthesis of large volumes of gold and silver nanoparticles

Alex Ross,[a,b] Marcelo Muñoz,[a] Benjamin H. Rotstein,[b,c] Erik J. Suuronen,[a] and Emilio I. Alarcon*[a,b]

[a]Division of Cardiac Surgery, University of Ottawa Heart Institute, Ottawa, ON, K1Y4W7, Canada
[b]Biochemistry, Microbiology and Immunology, University of Ottawa, Ottawa, ON, K1H8M5, Canada
[c]Molecular Imaging Probes and Radiochemistry Laboratory, University of Ottawa Heart Institute, 40 Ruskin Street, Ottawa, Canada
*email: ealarcon@ottawaheart.ca

Table of Contents

System parts 3

Table S1. System parts 3

3D printing specifications 4

System assembly and operation 5

System radiance 6

Table S2. Values of corresponding currents, voltages, and radiances 6

Figure S1. Absorbance spectra from 350 to 700 nm of uncapped gold nanoparticles made with different radiance intensities 7

System operating temperatures 8

Figure S2. Solution temperature in the quartz cell under irradiation over time 8

Anti-fouling experiments 9

Figure S3. Images of the quartz flow cell after 10 mL synthesis of various gold nanoparticle 9

Nanoparticle stability 10

Figure S4. Absorbance vs wavelength spectra for citrate capped and uncapped gold nanoparticles 10

Absorbance spectra 11

Figure S5. Representative absorption spectra for colloidal nanogold particles prepared with different capping agents 11

Figure S6. Representative absorption spectra for colloidal nanosilver particles prepared with different capping agents 12

TEM images 13

Figure S7. Representative TEM images for AuNP prepared in the presence of different capping agents 13

Figure S8. Representative TEM images for AgNP prepared in the presence of different capping agents 14

Silver nanoparticle size 15

Figure S9. Nanosilver TEM images for the different capping agents tested in this study. 15

CLKRS characterization 16

Figure S10. Mass Spectrometry for CLKRS 16

Statistical Analysis 17

Table S3. Student t-test for hydrodynamic sizes and zeta potential values of nanogold and nanosilver particles prepared using NPFloSS. 17

Table S4. Summary for mean sizes, standard deviation (SD), and polydispersity index (PDI) 20

Table S5. One-way ANOVA in KaleidaGraph 4.5 software for TEM images of nanogold particles prepared using NPFloSS. 21

Batch reproducibility 24

Table S6. Nanogold synthesis reproducibility 24

Table S7. One-way ANOVA of nanogold size counts. 24

Table S8. One-way ANOVA of nanogold size counts. 26

Video 27

S1. System parts

Table S1. List of system parts

PART	QUANTITY	NOTES
ARCTIC Alpine 64 GT Rev. 2 CPU Cooler	2	A heat sink with a block for drilling holes is needed to mount the LEDs
LZ4-V4UV0R-0000 high power 365 nm LED	2	The starboard is needed for mounting onto the heat sink
3D printed case	1	See 3D printing specifications for case
Peristaltic pump	1	The pump should support flow rates between 0.5 mL/min and 2 mL/min
Quartz cell	1	The cell used had an inner volume of 80 µL with physical dimensions of 41x6x4mm
Tubing	-	3 mm OD 1 mm ID rubber tubing was driven by the pump and used to connect the quartz cell and the 1 mm OD 0.5 mm ID PTFE tubing used for the rest of the system
Power supplies	2-4	Each pair of LEDs & fans can be powered together or individually for a total of 2-4 power units (12V 1 A to power the fans & 5A 30V for the LEDs). The LEDs should be operated with an adjustable power supply capable of delivering at least 15 watts to each LED
Thermal paste	1	Thermal paste must be applied to the space between the LED and heat sink to improve heat transfer
Electrical wiring	1	An electrical connection between the LED channels and the power supply is needed. The wiring code might vary depending on the LED model and manufacturer
Solder	1	The electrical wiring should be firmly soldered onto the board. The wiring of the fan is quite fragile and was thus soldered together with the wiring from the power source to improve resiliency
Screws (1 cm length 0.3 cm diameter)	4-6	Each LED must be securely mounted using 2-3 screws. The size of the screws should match the drill pattern made

S2. 3D printing specifications

The case file is accessible HERE as a .stl file. Ultimaker Cura was used to slice the .stl with the following characteristics:

- Profile: Engineering – Normal – 0.15
- Material: generic ABS
- Print core: AA 0.4
- Infill density: 100%
- Infill pattern: Triangles
- Layer height: 0.15 mm
- Wall thickness: 1.2 mm
- Printing temperature: 245°C
- Build plate temperature: 85°C
- Print speed: 30 mm/s
- Support: Everywhere (overhang angle 60)

An Ultimaker S5 was used to print with the following material profile:

- Material name: ABS
- Diameter: 2.85 mm
- Density: 1 kg/m^3

To improve adhesion, the bed was covered with a layer of glue before beginning printing.

S3. System assembly and operation (see tutorial videos, links in the last page)

ASSEMBLY (a step-by-step short tutorial is found in Video S1)

1. Unpackage and clean all materials. It is important that the quartz cell is cleaned with 10 mM HCl (overnight) and profusely rinsed with milli-Q water previous to assembling.
2. Place the LEDs on the surface of the heat sink metal blocks.
3. Using a marker, trace the starboard pattern of the LEDs and markdown 2-3 locations to drill holes for mounting.
4. Use a precision drill to make the holes for the screws.
5. Solder the necessary electrical connections for the LEDs and fans. In our case, the 4 channels of the LEDs were connected in parallel to a central wire to be connected to the power supply. For the fans, the two (out of four) pins corresponding to negative and positive were cut, stripped, and soldered to the wiring from a power supply so as to continuously run the fans at maximum output when plugged in.
6. Apply thermal paste and mount the LEDs onto the heat sink.
7. Insert the 2-3 screws per LED to secure the mount. Remove the excess of thermal paste using alcohol.
8. Place the case parts on each heat sink. With the fans facing down and the LEDs facing up, the circular holes for the tubing should be facing up and the rectangular hole for the wiring should be facing down and placed to allow the wiring to exit the case.
9. Connect the tubing to make a single flow from the input to the quartz cell to the output.
10. Insert the tubing into the peristaltic pump.
11. Place the tubing in the case so that the quartz cell is over the LED and the LED can be seen through the quartz cell when looking from above.
12. Place the second case part with the heat sink over the first part with the tubing inside. The holes should match to provide space for the tubing to pass through both cases and align them. The quartz cell should now be between two LEDs, one above and one below. If necessary, place some tape to securely hold the tubing connected to the quartz cell to the case in order to create an optimum position for light exposure.

OPERATION (a step-by-step short tutorial is found in Video S1)

1. Choose the desired LED power and flow rate. Running the LEDs at the maximum recommended operating power with the four LED channels connected in parallel resulted in a voltage of 4.2 V and a current of 2.8 A with our adjustable power supply. A flow rate of 0.5 mL/min for silver and 1.8 mL/min for gold was used.
2. Prime the system by running a 10 mM solution of Tween-20 in water through it. This can be done at any flow rate.
3. Turn on the LEDs and run the desired nanoparticle solution through with the appropriate flow rate. The LEDs do not have to be warmed up.
4. Clean the outer surface of the LED, when cold, and quartz cuvette with 70% ethanol. The quartz cell can be easily removed and cleaned with aqua regia if necessary. If cleaned with acid, discard the first mL of nanoparticles generated next run.

S4. System Radiance

To characterize the luminescent output of the system, the radiant output at 5 different evenly spaced power settings as well as the maximum recommended operating power was characterized (Table S2). Current and voltage data were taken from the display on the adjustable power source (1 unit powering 1 LED) whereas radiance was measured using a Luzchem L-0487 power meter with an attached Thorlabs 25 mm 2.0 neutral density filter placed at the usual position of the quartz cell while one LED was turned on at the given power. This radiance measure was multiplied by two to reflect the two LEDs present when the system is running.

Table S2. Values of corresponding currents, voltages, and radiances.

Current (A)	Voltage (V)	Radiance (mW/cm^2)
0.5	3.5	5.7
1.0	3.6	8.1
1.5	3.8	10.3
2.0	4.0	11.3
2.5	4.1	13.5
2.8	4.2	14.0

Greater radiant exposure should push a photochemical reaction closer to completion. Thus, the effect of 5 different increasing radiances for a standard flow (1.8 mL/min) was determined (Figure S1). As greater intensities seemed to produce better results, the maximum recommended operating current corresponding to a radiance of 14 mW/cm^2 was used for all subsequent runs.

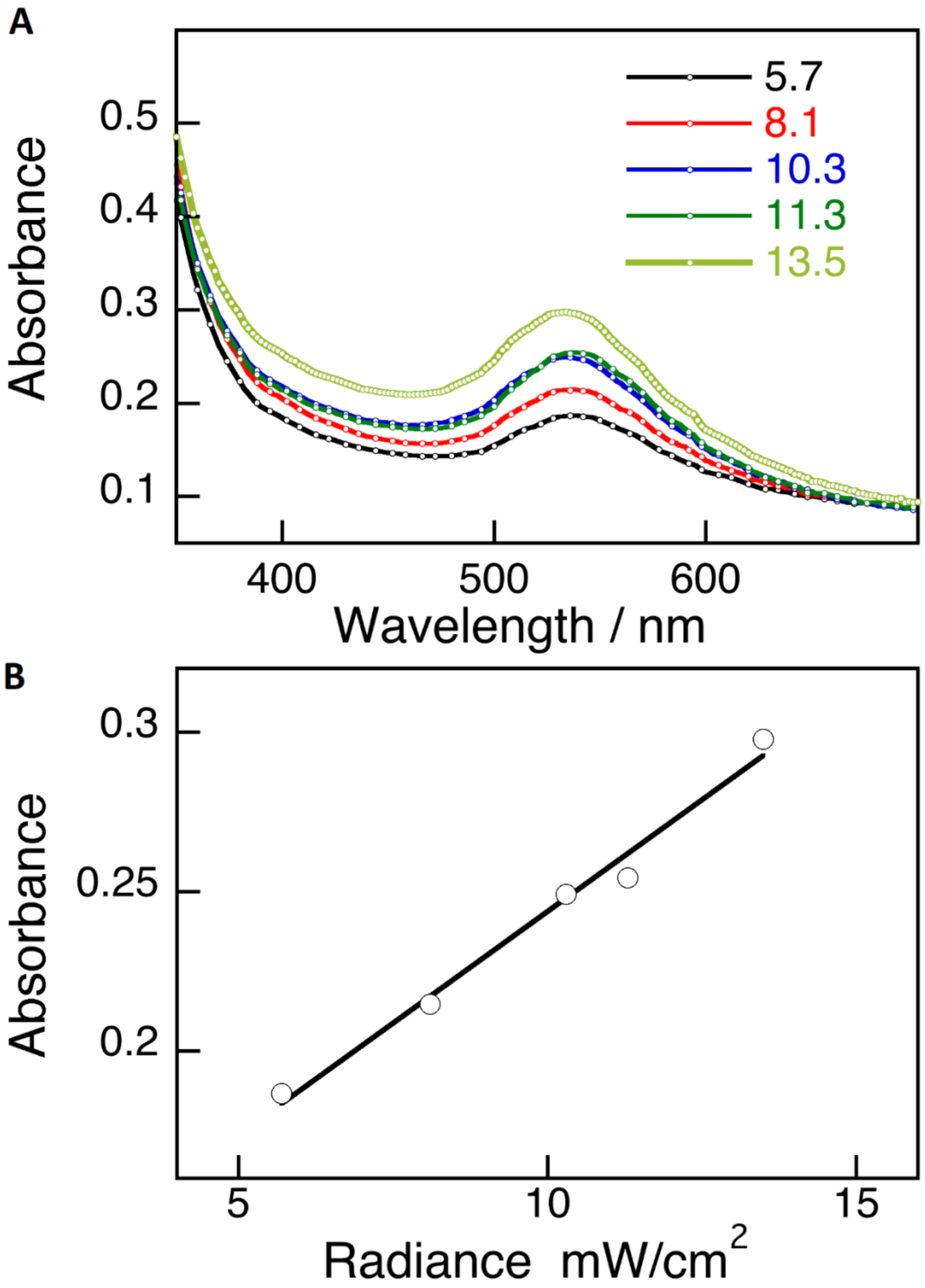

Figure S1. (A) Absorbance spectra from 350 to 700 nm of uncapped gold nanoparticles made with different radiance intensities. Spectra were immediately taken upon synthesis completion. Inset values are irradiance of the device (mW/cm^2) as presented in Table S2. **(B)** The absorbance value at the wavelength of maximal plasmonic absorbance (tau) for nanoparticles in **(A)**.

S5. System operating temperatures

The operating temperature of the LEDs was measured after reaching thermal equilibrium (run at 4.2 V 2.8A for 60 minutes with the temperature remaining constant since t=30 minutes) using a probe touching the starboard. The recorded equilibrium temperature was 33°C, far below the specified maximum operating temperature of 130°C.

The temperature of the solution being irradiated was also measured. Fresh water was run through the system to fill the quartz cell. The flow was then halted, and the LEDs were turned on at the standard 4.2 V 2.8A. After a given time period, the LEDs were turned off and the output tubing was quickly removed from the output side of the cell. Flow was resumed for about a second to pour solution out of the cell and onto a thermal probe. Results are displayed in Figure S2 below.

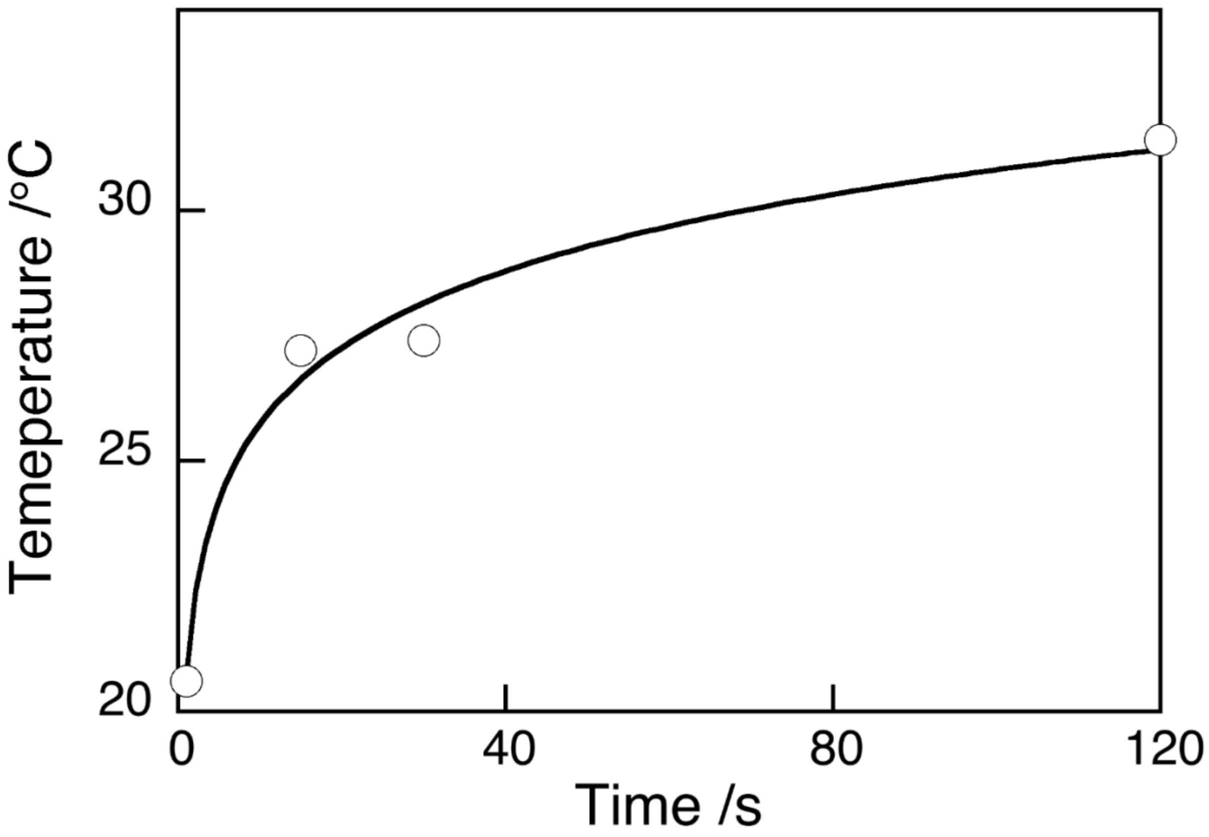

Figure S2. Solution temperature in the quartz cell under irradiation over time. Temperature of the solution was directly measured using a probe after a given irradiation time. Temperature values trended toward the system equilibrium temperature over time in a logarithmic manner.

S6. Anti-fouling experiments

Nanoparticle deposition on solid surfaces during flow synthesis is a significant obstacle to designing effective flow photoreactors. Deposition can be directly seen and leads to a loss of surface transparency, reducing reaction efficiency. Our approach to reduce reactor fouling is to add Tween-20 surfactant into the reactant solution. By adding increasing amounts of Tween-20, we were able to reduce fouling to a negligible amount that allowed for continuous reactor operation.

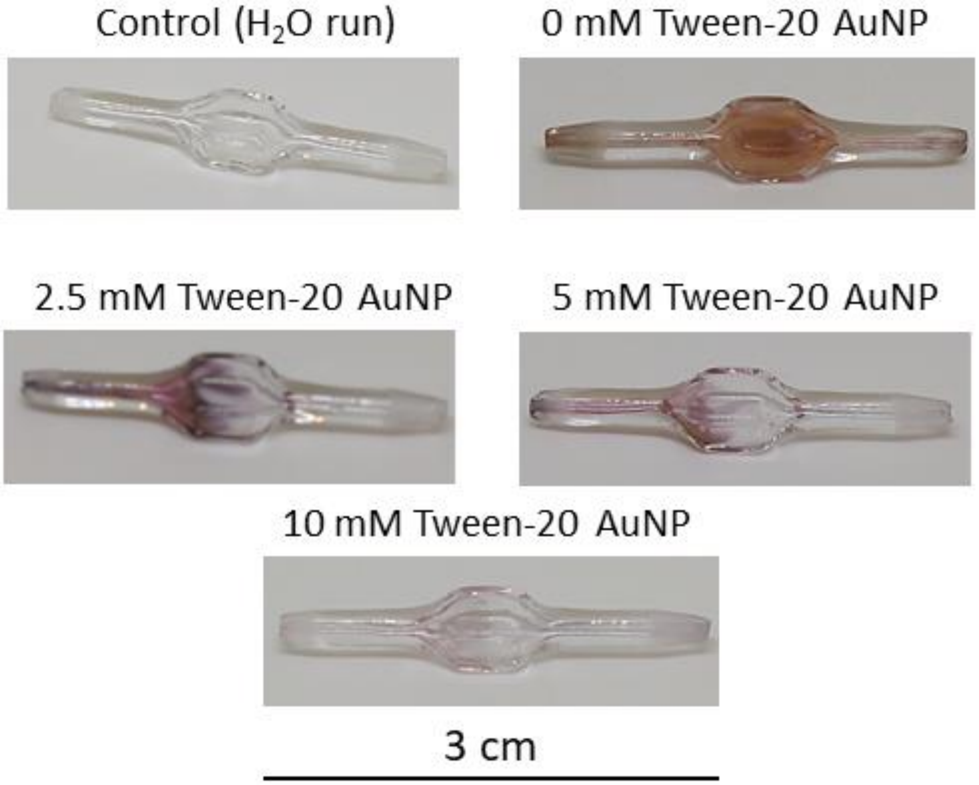

Figure S3. Images of the quartz flow cell after 10 mL synthesis of various gold nanoparticle solutions. Aqueous solutions of 1 mM $HAuCl_4$ along with 3 mM I-2959 were run with 0, 2.5, 5-, and 10-mM concentrations of Tween-20. A control run with pure water was also made. Increasing concentrations of Tween-20 decreased visible fouling of the system.

Attempts at controlling fouling by altering other variables such as flow rate and solution pH had negligible results (not shown) as the flow cell would appear fouled.

S7. Nanoparticle stability

The stability of generated nanoparticles was investigated as particle growth continued after synthesis. Thus, in general, particles were left to grow overnight before characterization unless otherwise specified. Two capping regiments, one with the good capping agent citrate and one with no capping agent were chosen to study the stability of particles generated by the system (Figure S3). Use of the good capping agent produced excellent stability while uncapped particles still showed good stability.

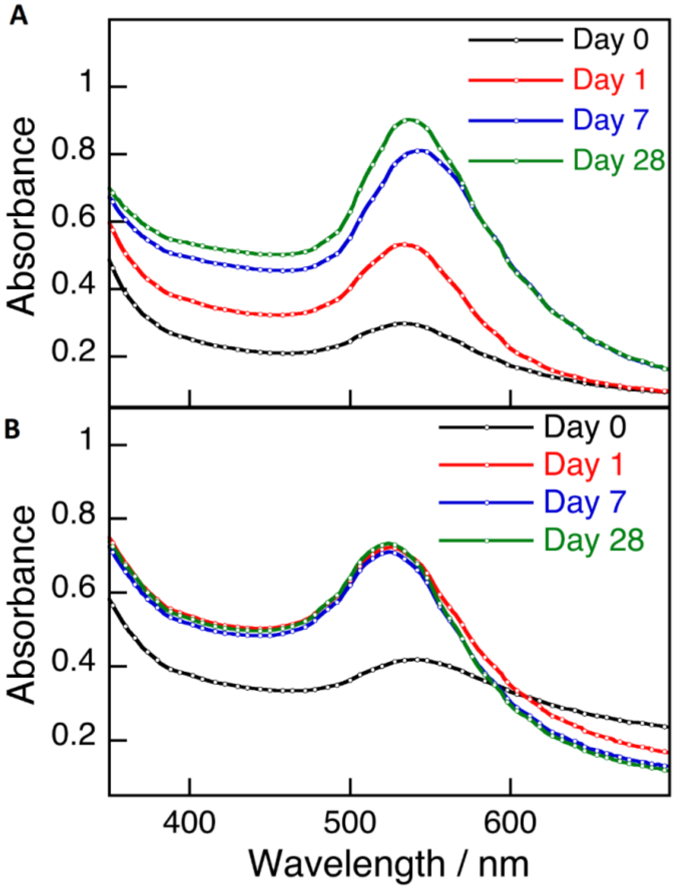

Figure S4. Absorbance vs wavelength spectra for **(A)** citrate capped and **(B)** uncapped gold nanoparticles. Samples were synthesized and then characterized by their absorbance from 350 to 700 nm immediately (day 0) and then 1, 7, and 28 days after.

S8. Absorption spectra for colloidal nanoparticles in the presence of different capping agents

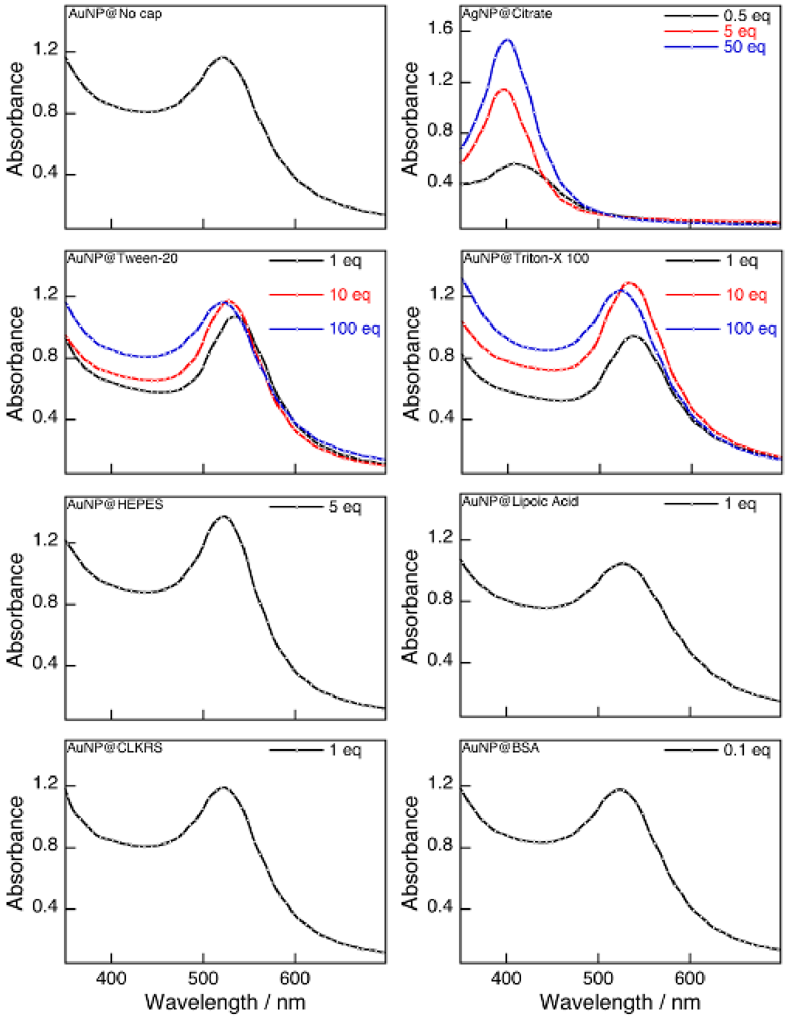

Figure S5. Representative absorption spectra for colloidal nanogold particles prepared with different capping agents as indicated in each plot. See main text for further details.

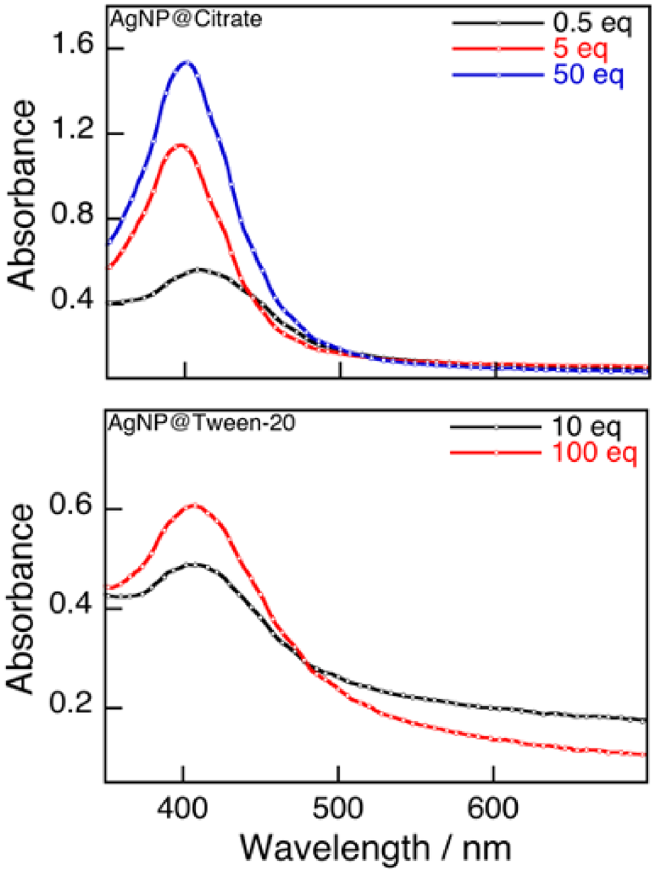

Figure S6. Representative absorption spectra for colloidal nanosilver particles prepared with different capping agents as indicated in each plot. See main text for further details.

S9. TEM images

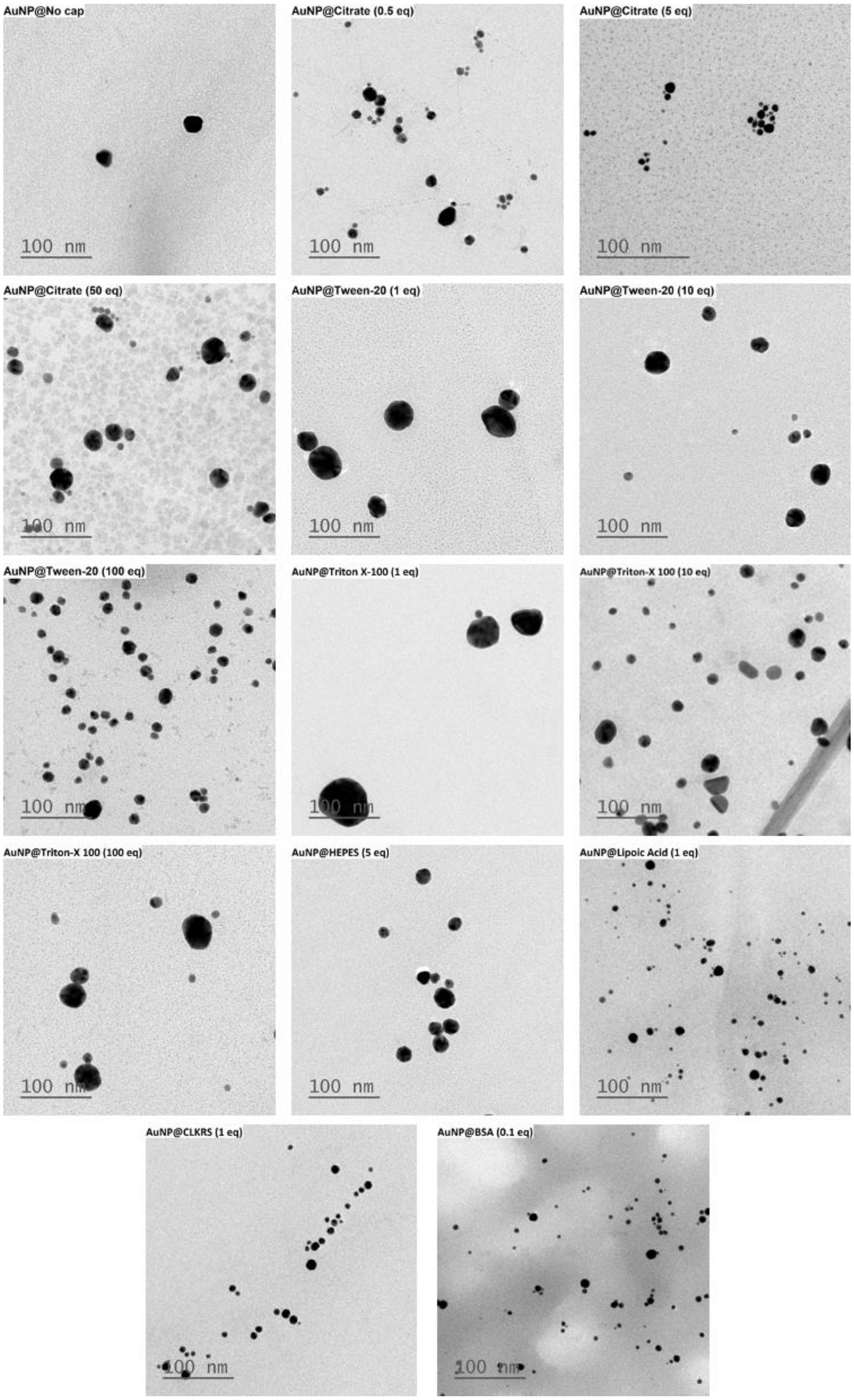

Figure S7. Representative TEM images for AuNP prepared in the presence of different capping agents as indicated in each image, see main text for further details. Scale bar corresponds to 100 nm in all cases.

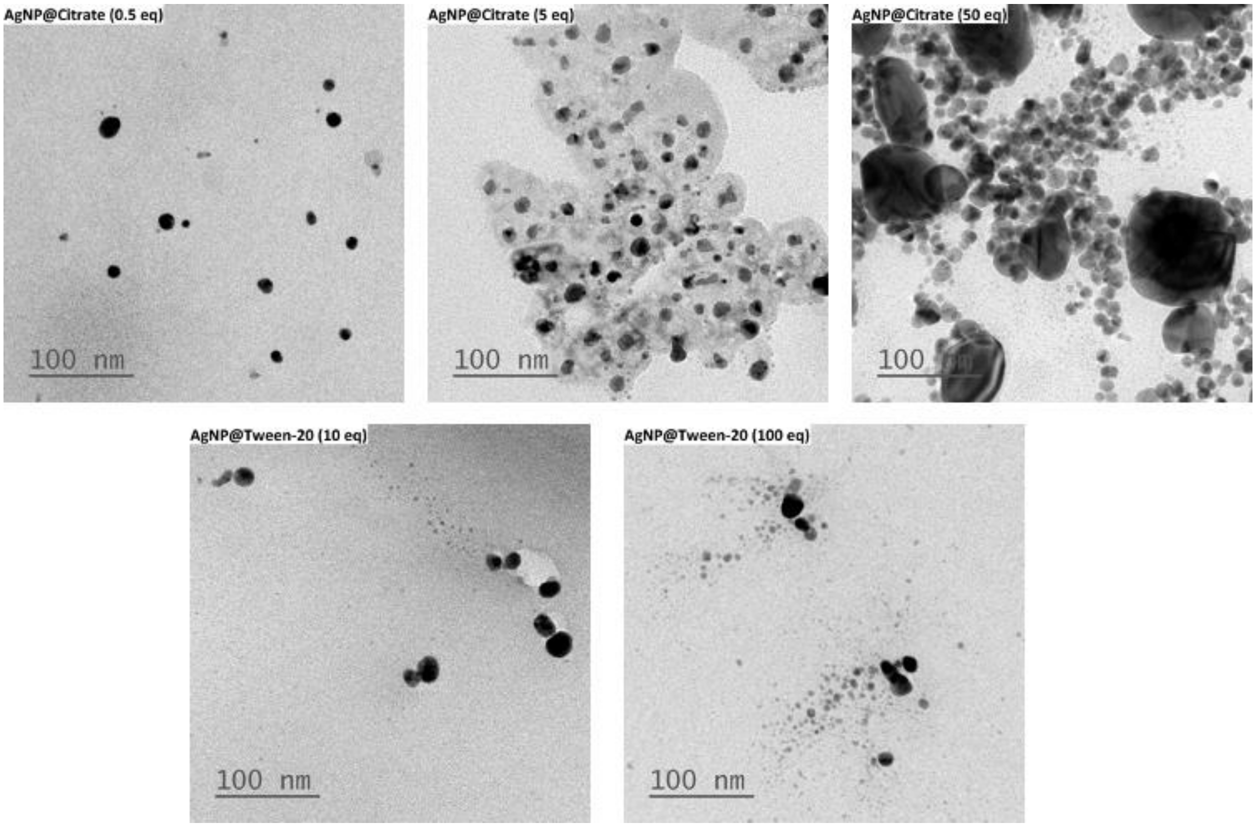

Figure S8. Representative TEM images for AgNP prepared in the presence of different capping agents as indicated in each image, see main text for further details. Scale bar corresponds to 100 nm in all cases.

S10. Silver nanoparticle size

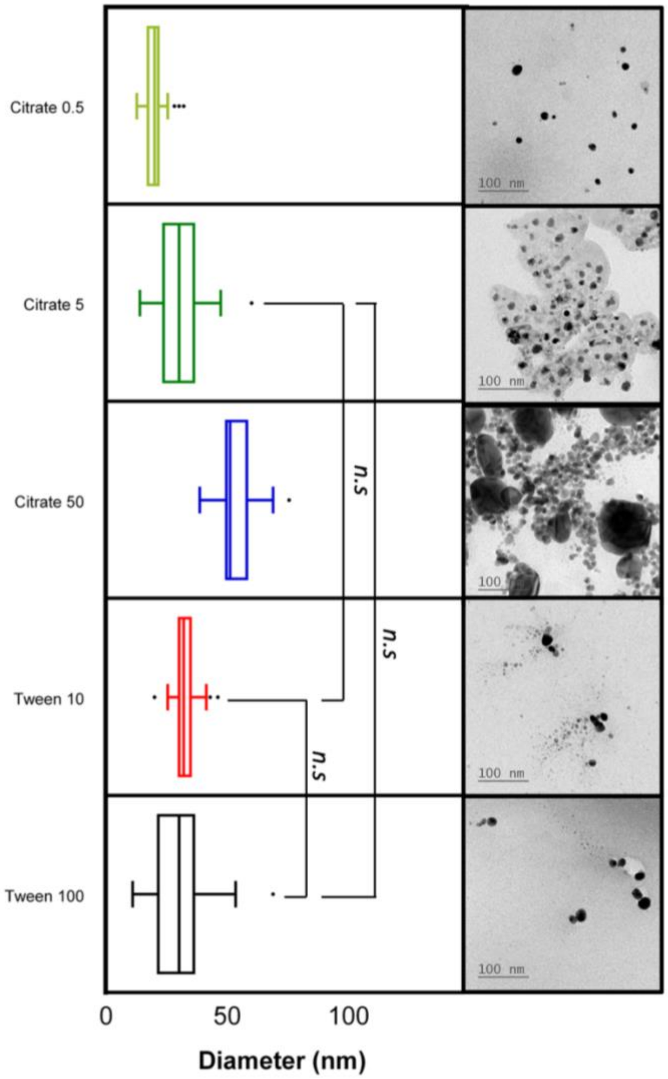

Figure S9. Nanosilver TEM images for the different capping agents tested in this study. Each histogram represents 100 individually measured nanoparticles. Representative TEM images of the nanoparticles are shown to the right of each histogram along with a 100 nm scale bar. Values in the Figure are represented as box plots where the box encloses 50% of the data, upper and lower quartile, with the median value of the variable displayed as a line inside the box. The bars extending from the top and bottom of each box mark the minimum and maximum values within the data set that fall within an acceptable range. P values are calculated by one-way ANOVA using Holm's multiple comparison analysis. ns in the Figure indicates no significant p values.

S11. CLKRS characterization

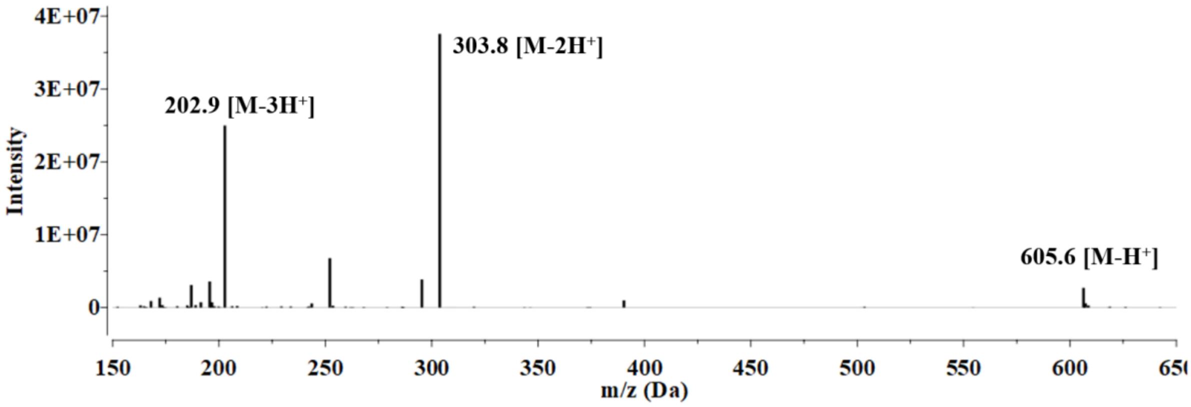

Figure S10. Mass Spectrometry for CLKRS. Mass Spectrometry analysis was performed in a Waters ACQUITY UPLC XEVO TQD with a column 2.1x100 mm BEH C8, with ESI in positive mode, and a flow rate of 0.5 mL/min, a gradient of 2% to 95% of Acetonitrile in 9 minutes, and constant 0.1% of Formic Acid.

S12. Statistical analysis

Table S3. Student t-test for hydrodynamic sizes and zeta potential values of nanogold and nanosilver particles prepared using NPFloSS.

Student t-test

**Nanoparticle (Property)**	**Comparison**	**p-value (0.05)**	**Significant?**
**GOLD**			
DLS	Tween 1 vs Tween 100	0.430852358	NO
DLS	Tween 10 vs Tween 100	0.430852358	NO
DLS	Tween 1 vs Tween 100	0.430852358	NO
DLS	Citrate 0.5 vs Citrate 5	0.09370309	NO
DLS	Citrate 0.5 vs Citrate 50	0.000167727	**YES**
DLS	Citrate 5 vs Citrate 50	0.093775535	NO
DLS	Triton 1 vs Triton 10	0.00199233	**YES**
DLS	Triton 1 vs Triton 100	0.127384362	NO
DLS	Triton 10 vs Triton 100	0.26022659	NO
DLS	Tween 10 vs uncapped	0.165259641	NO
DLS	Tween 10 vs HEPES 5	0.39250326	NO
DLS	Tween 10 vs Lipoic acid 1	0.59932311	NO
DLS	Tween 10 vs CLKRS 1	0.178815956	NO
DLS	Tween 10 vs BSA 0.5	0.771097837	NO
Zeta	Tween 1 vs Tween 100	0.00140761	**YES**
Zeta	Tween 10 vs Tween 100	0.001920268	**YES**
Zeta	Tween 1 vs Tween 100	0.00140761	**YES**
Zeta	Citrate 0.5 vs Citrate 5	0.170364806	NO
Zeta	Citrate 0.5 vs Citrate 50	0.000590205	**YES**
Zeta	Citrate 5 vs Citrate 50	0.65934158	NO
Zeta	Triton 1 vs Triton 10	0.1821556	NO

Zeta	Triton 1 vs Triton 100	0.043595671	**YES**
Zeta	Triton 10 vs Triton 100	0.015467514	**YES**
Zeta	Tween 10 vs uncapped	0.021343684	**YES**
Zeta	Tween 10 vs HEPES 5	0.179158812	NO
Zeta	Tween 10 vs Lipoic acid 1	0.41217252	NO
Zeta	Tween 10 vs CLKRS 1	0.130146091	NO
Zeta	Tween 10 vs BSA 0.5	0.000576834	**YES**
**SILVER**			
DLS	Tween 10 vs Tween 100	0.184949264	NO
DLS	Tween 10 vs CLKRS 1	0.872623216	NO
DLS	Citrate 0.5 vs Citrate 5	0.190934872	NO
DLS	Citrate 0.5 vs Citrate 50	0.297140546	NO
DLS	Citrate 5 vs Citrate 50	0.896996834	NO
Zeta	Tween 10 vs Tween 100	0.259325673	NO
Zeta	Tween 10 vs CLKRS 1	0.005657165	NO
Zeta	Citrate 0.5 vs Citrate 5	0.036557147	**YES**
Zeta	Citrate 0.5 vs Citrate 50	0.12991893	NO
Zeta	Citrate 5 vs Citrate 50	0.430852358	NO

Table S4. Summary for mean sizes, standard deviation (SD), and polydispersity index (PDI) measured from TEM images of nanogold and nanosilver particles prepared using NPFloSS.

**Nanogold**			
**Name**	**Mean**	**SD**	**PDI***
Uncapped	60.05590141	19.53354179	0.105791
Citrate 0.5	22.37911153	5.458347006	0.059489
Citrate 5	13.31805814	3.255778633	0.059762
Citrate 50	24.90412492	5.164040389	0.042997
Tween 1	45.75772192	9.101831814	0.039567
Tween 10	31.50451655	7.037643285	0.049901
Tween 100	26.05611407	4.343296826	0.027786
Triton 1	42.40097979	15.13094888	0.127345
Triton 10	27.78461196	11.57813727	0.173648
Triton 100	28.78218998	6.623660825	0.05296
HEPES	22.93799489	5.551663283	0.058578
Lipoic Acid	30.38275937	8.863565886	0.085106
CLKRS	42.65820175	16.50385089	0.149681
BSA	12.79112845	4.136332658	0.104571
**Nanosilver**			
Citrate 0.5	20.47720008	3.666919473	0.032067
Citrate 5	30.76664017	8.04134023	0.068312
Citrate 50	53.54419691	6.792463347	0.016093
Tween 10	32.41123796	4.127253876	0.016216
Tween 100	30.16156443	9.868253992	0.107047
CLKRS	9.096169572	3.759377596	0.170811

*Nanoparticle polydispersity were estimated using $PDI=(SD/mean)^2$.

Table S5. One-way ANOVA in KaleidaGraph 4.5 software for TEM images of nanogold particles prepared using NPFloSS.

**Analysis of Variance Results**					
**Source**	DF	SS	MS	F	P
Total	1193	230404.45	193.1303		
A	13	133858.81	10296.832	125.84993	< .0001
Error	1180	96545.636	81.818335		
**Holm's Multiple Comparison**					
**Comparison**	**Mean Difference**	**|t|**	**2-P**	**adjusted Alpha**	**Significant?**
Tween 1 vs Triton 10	17.9731	12.2307	0	0.0005495	**YES**
Tween 1 vs Tween 100	19.7016	13.4069	0	0.0005556	**YES**
Tween 1 vs Tween 10	14.2532	9.6993	0	0.0005618	**YES**
Tween 1 vs Triton 100	16.9755	11.5518	0	0.0005682	**YES**
Tween 1 vs BSA	32.9666	22.4337	0	0.0005747	**YES**
Tween 1 vs Lipoic Acid	15.375	10.4626	0	0.0005814	**YES**
Tween 1 vs HEPES	22.8197	15.5288	0	0.0005882	**YES**
Citrate 50 vs BSA	12.113	9.4692	0	0.0005952	**YES**
Citrate 5 vs Lipoic Acid	-17.0647	11.7853	0	0.0006024	**YES**
Citrate 5 vs Triton 100	-15.4641	10.6799	0	0.0006098	**YES**
Citrate 5 vs Triton 10	-14.4666	9.991	0	0.0006173	**YES**
Citrate 5 vs CLKRS	-29.3401	20.2631	0	0.000625	**YES**
Citrate 50 vs CLKRS	-17.7541	13.879	0	0.0006329	**YES**
Citrate 50 vs Triton 1	-17.4969	9.0471	0	0.000641	**YES**
Citrate 50 vs Tween 1	-20.8536	14.1908	0	0.0006494	**YES**
Triton 100 vs BSA	15.9911	12.5008	0	0.0006579	**YES**
Triton 100 vs CLKRS	-13.876	10.8474	0	0.0006667	**YES**
Triton 10 vs BSA	14.9935	11.7209	0	0.0006757	**YES**
HEPES vs CLKRS	-19.7202	15.416	0	0.0006849	**YES**
CLKRS vs BSA	29.8671	23.3481	0	0.0006944	**YES**

Lipoic Acid vs BSA	17.5916	13.752	0	0.0007042	**YES**
Lipoic Acid vs CLKRS	-12.2754	9.5961	0	0.0007143	**YES**
Triton 10 vs CLKRS	-14.8736	11.6272	0	0.0007246	**YES**
Tween 100 vs Triton 1	-16.3449	8.4514	0	0.0007353	**YES**
Tween 10 vs BSA	18.7134	14.6289	0	0.0007463	**YES**
Tween 10 vs CLKRS	-11.1537	8.7192	0	0.0007576	**YES**
Tween 100 vs CLKRS	-16.6021	12.9784	0	0.0007692	**YES**
Triton 1 vs BSA	29.6099	15.3103	0	0.0007812	**YES**
Triton 1 vs HEPES	19.463	10.0637	0	0.0007937	**YES**
Tween 100 vs BSA	13.265	10.3697	0	0.0008065	**YES**
Citrate 0.5 vs Triton 1	-20.0219	10.3527	0	0.0008197	**YES**
Uncapped vs Triton 10	32.2713	19.2386	0	0.0008333	**YES**
Citrate 0.5 vs Tween 1	-23.3786	15.9091	0	0.0008475	**YES**
Uncapped vs Tween 100	33.9998	20.269	0	0.0008621	**YES**
Citrate 0.5 vs CLKRS	-20.2791	15.8529	0	0.0008772	**YES**
Uncapped vs Citrate 5	46.7378	25.8304	0	0.0008929	**YES**
Uncapped vs HEPES	37.1179	22.1279	0	0.0009091	**YES**
Uncapped vs CLKRS	17.3977	10.3717	0	0.0009259	**YES**
Uncapped vs Lipoic Acid	29.6731	17.6897	0	0.0009434	**YES**
Uncapped vs Citrate 50	35.1518	20.9558	0	0.0009615	**YES**
Uncapped vs Triton 100	31.2737	18.6439	0	0.0009804	**YES**
Uncapped vs BSA	47.2648	28.177	0	0.001	**YES**
Citrate 5 vs Tween 10	-18.1865	12.56	0	0.00102	**YES**
Citrate 5 vs Triton 1	-29.0829	14.1902	0	0.001042	**YES**
Citrate 5 vs Tween 100	-12.7381	8.7972	0	0.001064	**YES**
Uncapped vs Citrate 0.5	37.6768	22.4611	0	0.001087	**YES**
Citrate 5 vs Tween 1	-32.4397	20.0425	0	0.001111	**YES**
Uncapped vs Tween 10	28.5514	17.021	0	0.001136	**YES**
Citrate 5 vs Citrate 50	-11.5861	8.0016	2.89E-15	0.001163	**YES**

Uncapped vs Triton 1	17.6549	7.9614	4.00E-15	0.00119	**YES**
HEPES vs BSA	10.1469	7.9322	4.89E-15	0.00122	**YES**
Uncapped vs Tween 1	14.2982	7.8273	1.11E-14	0.00125	**YES**
Triton 1 vs Triton 10	14.6164	7.5577	8.22E-14	0.001282	**YES**
Citrate 0.5 vs BSA	9.58798	7.4953	1.30E-13	0.001316	**YES**
Citrate 0.5 vs Tween 10	-9.12541	7.1337	1.70E-12	0.001351	**YES**
Triton 1 vs Triton 100	13.6188	7.0419	3.21E-12	0.001389	**YES**
Tween 10 vs HEPES	8.56652	6.6968	3.29E-11	0.001429	**YES**
Citrate 5 vs HEPES	-9.61994	6.6438	4.66E-11	0.001471	**YES**
Citrate 0.5 vs Citrate 5	9.06105	6.2578	5.45E-10	0.001515	**YES**
Citrate 0.5 vs Lipoic Acid	-8.00365	6.2567	5.49E-10	0.001562	**YES**
Triton 1 vs Lipoic Acid	12.0182	6.2143	7.14E-10	0.001613	**YES**
HEPES vs Lipoic Acid	-7.44476	5.8198	7.58E-09	0.001667	**YES**
Tween 10 vs Triton 1	-10.8965	5.6342	2.20E-08	0.001724	**YES**
Citrate 50 vs Tween 10	-6.60039	5.1598	2.90E-07	0.001786	**YES**
Citrate 0.5 vs Triton 100	-6.40308	5.0055	6.42E-07	0.001852	**YES**
Triton 100 vs HEPES	5.84419	4.5686	5.43E-06	0.001923	**YES**
Citrate 50 vs Lipoic Acid	-5.47863	4.2828	2.00E-05	0.002	**YES**
Tween 10 vs Tween 100	5.4484	4.2592	2.22E-05	0.002083	**YES**
Citrate 0.5 vs Triton 10	-5.4055	4.2257	2.57E-05	0.002174	**YES**
Triton 10 vs HEPES	4.84662	3.7888	0.000159	0.002273	**YES**
Tween 100 vs Lipoic Acid	-4.32665	3.3823	0.0007424	0.002381	**YES**
Citrate 50 vs Triton 100	-3.87807	3.0316	0.002485	0.0025	**YES**
Tween 10 vs Triton 10	3.7199	2.908	0.003706	0.002632	**NO**
Citrate 0.5 vs Tween 100	-3.677	2.8744	0.00412		**NO**
Tween 100 vs HEPES	3.11812	2.4375	0.01493		**NO**
Citrate 50 vs Triton 10	-2.88049	2.2518	0.02452		**NO**
Tween 100 vs Triton 100	-2.72608	2.1311	0.03329		**NO**
Tween 10 vs Triton 100	2.72233	2.1281	0.03353		**NO**

Tween 1 vs CLKRS	3.09952	2.1092	0.03514		**NO**
Triton 10 vs Liooic Acid	-2.59815	2.0311	0.04247		**NO**
Citrate 0.5 vs Citrate 50	-2.52501	1.9739	0.04863		**NO**
Tween 1 vs Triton 1	3.35674	1.6257	0.1043		**NO**
Citrate 50 vs HEPES	1.96613	1.537	0.1246		**NO**
Tween 100 vs Triton 10	-1.7285	1.3512	0.1769		**NO**
Triton 100 vs Lipoic Acid	-1.60057	1.2512	0.2111		**NO**
Citrate 50 vs Tween 100	-1.15199	0.9006	0.368		**NO**
Tween 10 vs Lipoic Acid	1.12176	0.8769	0.3807		**NO**
Triton 10 vs Triton 100	-0.997578	0.7798	0.4356		**NO**
Citrate 0.5 vs HEPES	-0.558884	0.4369	0.6623		**NO**
Citrate 5 vs BSA	0.52693	0.3639	0.716		**NO**
Triton 1 vs CLKRS	-0.257222	0.133	0.8942		**NO**

S12. Batch reproducibility

To assess reproducibility, three batches of AuNP capped with citrate (5 eq) were synthesized. DLS and TEM measurements of particle diameter were taken at 1 day and 7 days after synthesis for each batch. Mean particle diameter is reported in nm.

Table S6. Nanogold synthesis reproducibility. measured in three independent batches at different days post-synthesis. Measurements were carried out using DLS or TEM.

**Batch**	**1**	**2**	**3**
Size (nm) DLS, Day 1	16.19±1.05	15.31±0.71	15.98±3.03
Size (nm) DLS, Day 7	19.04±0.55	18.91±0.48	17.88±0.34
Size (nm) TEM, Day 1	13.6±5.15	13.3±3.3	14.1±5.2
Size (nm) TEM, Day 7	14.6±5.22	13.7±3.1	14.8±3.8

Particles from several TEM images per batch were sized. Raw data of particle size (diameter) counts are available in our Figshare file, see main text for details. TEM values were obtained from counting 30-100 individual nanoparticles. One-way ANOVA using Holm's multiple comparison is included below.

Table S7. One-way ANOVA of nanogold size counts. Analysis was performed in KaleidaGraph 4.5 software for TEM images of nanogold particles prepared using NPFloSS in the presence of 5eq of citrate.

Analysis of Variance Results					
Source	DF	SS	MS	F	P
Total	451	7481.7091	16.589155		
A	5	112.12008	22.424017	1.3570786	0.23936
Error	446	7369.589	16.523742		

**Holm's Multiple Comparison**					
Comparison	Mean Difference	|t|	2-P	adjusted Alpha	Significant?
Batch 2-Day 1 vs Batch 3-Day 7	-1.52214	2.0464	0.0413	0.003333	**NO**
Batch 2-Day 1 vs Batch 1-Day 7	-1.23835	1.9031	0.05767		**NO**
Batch 2-Day 7 vs Batch 3-Day 7	-1.1151	1.6436	0.101		**NO**
Batch 1-Day 7 vs Batch 2-Day 7	0.831309	1.4461	0.1489		**NO**
Batch 1-Day 1 vs Batch 3-Day 7	-1.24081	1.3775	0.1691		**NO**

Batch 2-Day 1 vs Batch 3-Day 1	-0.78195	1.2017	0.2301	**NO**
Batch 1-Day 1 vs Batch 1-Day 7	-0.957023	1.1592	0.247	**NO**
Batch 3-Day 1 vs Batch 3-Day 7	-0.740193	1.091	0.2759	**NO**
Batch 3-Day 1 vs Batch 1-Day 7	-0.456402	0.7939	0.4277	**NO**
Batch 3-Day 1 vs Batch 2-Day 7	0.374907	0.6522	0.5146	**NO**
Batch 2-Day 1 vs Batch 2-Day 7	-0.407043	0.6255	0.5319	**NO**
Batch 1-Day 1 vs Batch 3-Day 1	-0.50062	0.6064	0.5446	**NO**
Batch 1-Day 7 vs Batch 3-Day 7	-0.28379	0.4183	0.6759	**NO**
Batch 1-Day 1 vs Batch 2-Day 1	0.28133	0.3197	0.7494	**NO**
Batch 1-Day 1 vs Batch 2-Day 7	-0.125714	0.1523	0.879	**NO**

Table S8. One-way ANOVA in KaleidaGraph 4.5 software for TEM images of nanosilver particles prepared using NPFloSS.

Analysis of Variance Results

Source	DF	SS	MS	F	P
**Total**	499	82743.81	165.81926		
**A**	4	59116.107	14779.027	309.62038	< .0001
**Error**	495	23627.703	47.732733		

Holm's Multiple Comparison

Comparison	Mean Difference	|t|	2-P	adjusted Alpha	Significant?
Citrate 5 vs Citrate 50	-22.7776	23.3122	0	0.005	**YES**
Citrate 50 vs Tween 10	21.133	21.629	0	0.005556	**YES**
Citrate 50 vs Tween 100	23.3826	23.9315	0	0.00625	**YES**
Citrate 0.5 vs Tween 100	-9.68436	9.9117	0	0.007143	**YES**
Citrate 0.5 vs Citrate 50	-33.067	33.8432	0	0.008333	**YES**
Citrate 0.5 vs Citrate 5	-10.2894	10.531	0	0.01	**YES**
Citrate 0.5 vs Tween 10	-11.934	12.2142	0	0.0125	**YES**
Tween 10 vs Tween 100	2.24967	2.3025	0.02172	0.01667	**YES**
Citrate 5 vs Tween 10	-1.6446	1.6832	0.09297		**YES**
Citrate 5 vs Tween 100	0.605076	0.6193	0.536		**YES**
Citrate 5 vs Citrate 50	-22.7776	23.3122	0	0.005	**YES**
Citrate 50 vs Tween 10	21.133	21.629	0	0.005556	**YES**
Citrate 50 vs Tween 100	23.3826	23.9315	0	0.00625	**YES**
Citrate 0.5 vs Tween 100	-9.68436	9.9117	0	0.007143	**YES**
Citrate 0.5 vs Citrate 50	-33.067	33.8432	0	0.008333	**YES**
Citrate 0.5 vs Citrate 5	-10.2894	10.531	0	0.01	**YES**
Citrate 0.5 vs Tween 10	-11.934	12.2142	0	0.0125	**YES**
Tween 10 vs Tween 100	2.24967	2.3025	0.02172	0.01667	**NO**
Citrate 5 vs Tween 10	-1.6446	1.6832	0.09297		**NO**
Citrate 5 vs Tween 100	0.605076	0.6193	0.536		**NO**

S13. Video

A short step by step guide for assembling and operation of the nanoparticle flow reactor. Video can be accessed in this link:
https://www.dropbox.com/s/vfchdgu8wp9znwo/video%20flow%20reactor.m4v?dl=0
